# Factors associated with prescription of modern antidiabetics in newly diagnosed patients with type 2 diabetes. a real-world data study in a Spanish region

**DOI:** 10.3389/fphar.2025.1530139

**Published:** 2025-07-11

**Authors:** Irantzu Bengoa-Urrengoechea, Isabel Aguilar-Palacio, María José Rabanaque, María Jesús Lallana, Adriana Gamba Cabezas, Sara Malo

**Affiliations:** ^1^ Lozano Blesa University Clinical Hospital, Zaragoza, Spain; ^2^ Group in Health Services in Aragón (GRISSA), Fundación Instituto de Investigación Sanitaria de Aragón (IIS Aragón), Zaragoza, Spain; ^3^ Department of Preventive Medicine and Public Health, University of Zaragoza, Zaragoza, Spain; ^4^ Network for Research on Chronicity, Primary Care, and Health Promotion (RICAPPS), Instituto de Salud Carlos III (ISCIII), Madrid, Spain; ^5^ Aragon Health Service, Zaragoza, Spain

**Keywords:** antidiabetic drug, type 2 diabetes, prescribing pattern, multilevel analysis, real word data

## Abstract

**Aim:**

To describe the patterns of first prescription of antidiabetic drugs (AD) in patients with type 2 diabetes (T2D) and analyze the factors associated with the prescription of a modern one.

**Methods:**

Observational longitudinal study conducted in the CArdiovascular Risk factors for HEalth Services research (CARhES) cohort. Individuals older than 15, resident in Aragón (Spain), diagnosed with T2D during 2018–2022 were selected and followed-up until 31^st^ December 2022. Secondary use of data from the health system provided sociodemographic, clinical and pharmacological prescription information. We also considered additional variables by Basic Healthcare Area (BHA) of residence. AD were classified into “classical” and “modern” and their differences were described and compared. A multilevel methodology stratified by sex was developed, considering individual characteristics and characteristics of the BHA of residence, to analyze the factors associated to a modern AD.

**Results:**

Our population-based cohort of 22,892 patients were mostly male, native, low-income and living in non-depopulated BHA. People who were younger, with heart failure, ischemic heart disease, chronic renal failure, obesity, with a previous major adverse cardiovascular event, higher socioeconomic level or lived in less deprived and more depopulated areas were more likely to get a modern AD prescription.

**Conclusion:**

Our analyses showed that prescribing practices vary according to a range of sociodemographic, clinical and geographical characteristics. Knowledge of these factors is essential for implementing and improving equitable and person-centered approaches.

## 1 Introduction

The prevalence of diabetes is increasing at an unstoppable rate (Internacional Diabetes Federation, 2023). Type 2 diabetes (T2D) accounts for the vast majority (over 90%) of diabetes worldwide. Upon diagnosis of T2D, it is common practice to recommend hypoglycemic treatment in conjunction with lifestyle modification (Alemán et al., 2018). The timeliness of this treatment should be assessed by the physician based on the patient’s comorbidities and analytical results (mainly fasting plasma glucose (FPG), glycosylated hemoglobin (HbA1c) and glomerular filtration rate (GFR)) in order to select the most appropriate treatment for each patient.

The increasing availability of antidiabetic drugs (AD) for the management of T2D currently allows different options for achieving glycemic targets and choosing the therapy that best suits the individual characteristics of each patient (Dahlén et al., 2022; DeMarsilis et al., 2022). AD can be classified as “modern” or “classical” based on their mechanism of action. “Classical” correspond to those whose mechanism is based on β-cell dysfunction and insulin resistance (metformin, sulfonylureas, alpha-glucosidase inhibitors, thiazolidinediones, repaglinide, insulins) and “modern” those with an incretin effect and with a hypoglycaemic action at renal level (Dipeptidyl peptidase four inhibitors (DPP4i), Sodium-Glucose Transport Protein 2 (SGLT2) inhibitors (SGLT2i) and glucagon-like peptide-1 agonists (GLP1a)). After the commercialization of new drugs, especially SGLT2i and GLP1a, research analyzing their effectiveness and safety has demonstrated the increased benefits obtained *versus* the classical therapy, especially in individuals with certain comorbidities such as cardiovascular (CV) disease, chronic kidney disease or heart failure (Herrington et al., 2023; van der Aart-van der Beek et al., 2022; Palmer et al., 2021; Ussher and Drucker, 2023). These drugs have been shown to reduce CV mortality, slow the progression of renal impairment, and reduce the frequency of hospital readmissions for heart failure (McDonagh et al., 2022). Because of this, they have been gaining prominence as first-line drugs in certain pathologies and clinical conditions, which has been reflected in the evolution of treatment algorithms (Alemán et al., 2014; Mata et al., 2020; García Soidán, 2023).

It is necessary to analyze whether, following the introduction of these new drugs with new indications, they have become a reference in routine clinical practice. Furthermore, it is important to identify the factors associated with their prescription. In addition to the clinical characteristics of the patients and their analytical values, factors such as their socioeconomic level or geographical location must also be considered, in order to explore the existence of variations in clinical practice.

The objective of this study is to describe the pattern of AD prescription in newly diagnosed patients with T2D and to analyze the factors associated with the prescription of a modern AD.

## 2 Materials and methods

### 2.1 Study design and population

We conducted a retrospective, longitudinal, observational study based on the CARhES Cohort (CArdiovascular Risk factors for HEalth Services research), a dynamic cohort that collects information on patients with at least one CV risk factor (hypertension, T2D or dyslipidemia) in Aragón and was launched from 2017. The CARhES cohort was approved by the Research Ethics Committee of Aragón (CEICA, PI21/148) (Aguilar-Palacio et al., 2024). Aragon is located in the north-east of Spain and had around 1.3 million inhabitants in 2018. The Spanish health system is mainly tax financed and is based on universality, free healthcare, equity and fairness in funding (Bernal-Delgado et al., 2024).

**FIGURE 1:**
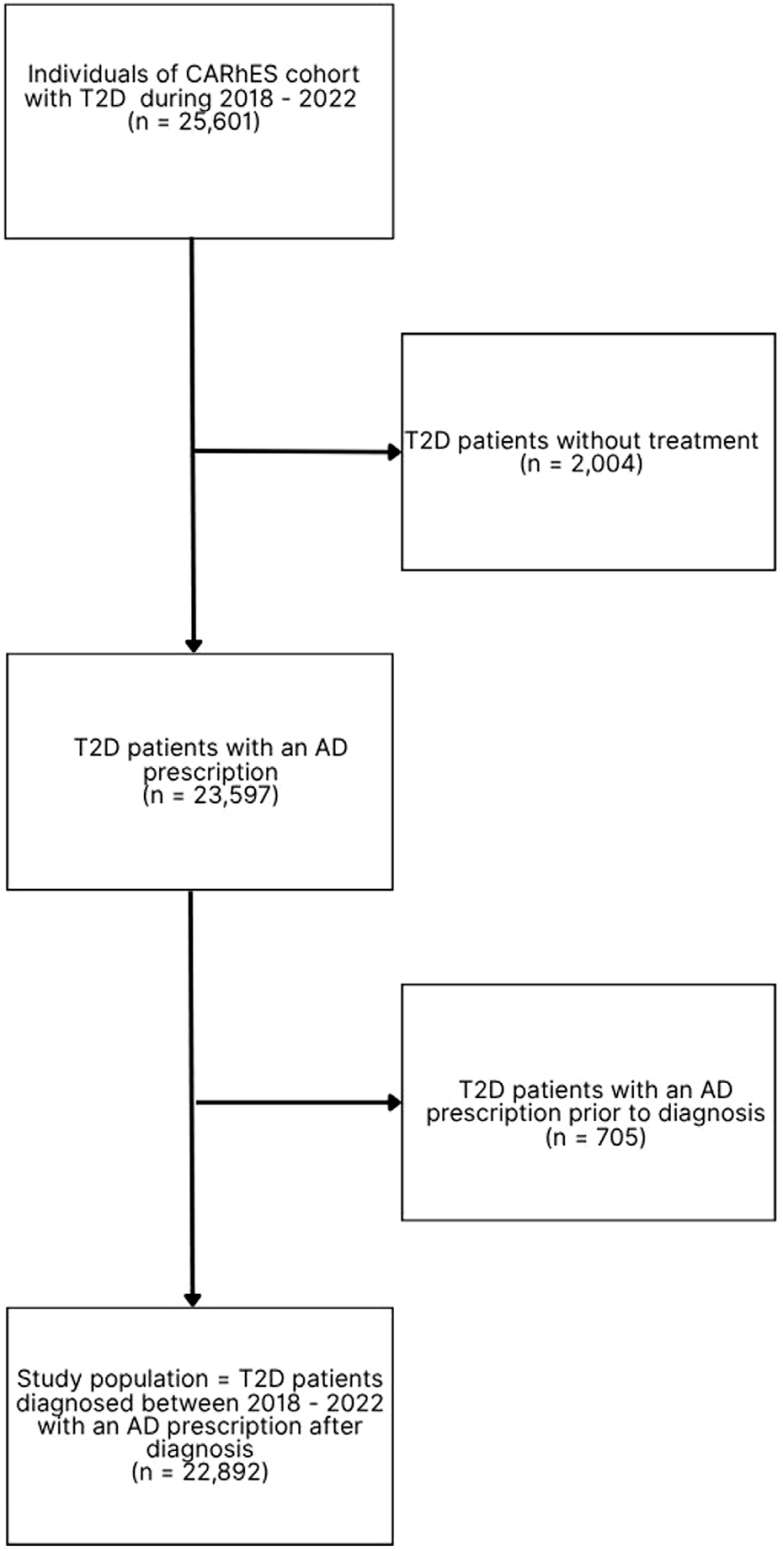
Flow chart of the study population. n: number of patients; T2D: type 2 diabetes; AD: antidiabetic drug.

For the present study, all the individuals of the CARhES cohort aged ≥16 years old with a new diagnosis of T2D between 1 January 2018 and 31 December 2022 were selected. The diabetes diagnosis was identified by a new diagnosis in Primary Care (PC), an emergency department or hospital, or with the first prescription of an AD. All patients with a first AD prescription prior to the diagnosis of T2D and those without treatment were excluded. The flow chart of the study population is shown in Figure 1.

### 2.2 Variables of the study and data sources

Sociodemographic and clinical characteristics of all the individuals in the cohort were described. Regarding sociodemographic characteristics, we considered sex, age (categorized into 15–44, 45–64, 65–79 and 80 years or older), immigrant status (native/immigrant) and socioeconomic level. Socioeconomic level was calculated on the basis of pharmacy copayment levels and the type of economic activity, according to the type of user of the Aragón health service. From the combination of these two variables, six mutually exclusive categories were obtained (employed individuals earning less than 18,000 € per year, employed individuals earning 18,000 € per year or more, individuals receiving the unemployment allowance, individuals with a contributory pension of less than 18,000 € per year and individuals receiving free medicines (people with minimum integration income or who no longer receive the unemployment allowance), individuals with a contributory pension of 18,000 € per year or more, and others). The clinical information included was obtained from the adjusted morbidity groups (GMA) (Barrio-Cortes et al., 2020). This source of information considers all medical diagnoses available in primary healthcare, emergencies department and hospital discharge records. The morbidity burden is a summary index included in the GMA database. It is based on the clinical conditions present in the patient and the weight assigned to each condition according to the care and resources needed for its management. It was originally developed with data from the Spanish health system. From electronic medical records we collected major adverse CV events (MACE; myocardial infarction, stroke and CV death) and laboratory variables (FPG, HbA1c, GFR). Treatment data was obtained by collecting the ATC code(s) (A10) corresponding to the first prescription date as described in Supplementary Appendix S1.

Treatments were classified into “classical” and “modern”. “Classical” correspond to those whose mechanism is based on β-cell dysfunction and insulin resistance (ATC codes: A10BA2 (metformin), A10BB (sulfonylureas), A10BD (0–6) (combinations of oral blood AD), A10BF (alpha-glucosidase inhibitors), A10BG (thiazolidinediones), A10BX (repaglinide), A10A (insulins)) and “modern” those with an incretin effect and with a hypoglycemic action at renal level (ATC codes: A10BD (Palmer et al., 2021; Ussher and Drucker, 2023; McDonagh et al., 2022; Alemán et al., 2014; Mata et al., 2020; García Soidán, 2023; Aguilar-Palacio et al., 2024; Bernal-Delgado et al., 2024; Barrio-Cortes et al., 2020; Compés Dea et al., 2018; Spanish ministry, 2023; Bates et al., 2015; Moreno-Juste et al., 2020; Mata-Cases et al., 2016; Rojo-Martínez et al., 2020; Wang et al., 2019; Ali et al., 2022; Choe et al., 2018) (combinations of oral blood AD), A10BH (DPP4i), A10BJ (GLP1a), A10BK (SGLT2i)).

Sociodemographic and clinical variables were collected at the time of entry into the cohort with a T2D diagnosis. The recording of the variable obesity is not uniform, so that obesity at any time was taken to mean that they were obese. Analytical variables were collected at the closest time prior to the first prescription of AD (from 12 months before or, if this was not possible, up to 1 month after).

We also considered three additional variables related to the Basic Healthcare Area (BHA) of residence of each patient. The first variable was the BHA deprivation index categorized into four quartiles, from least (Q1) to highest (Q4) deprived. This deprivation index combines information of four indicators from the Population and Housing Census 2011 (last index available) (Compés Dea et al., 2018). Other variable obtained by BHA was the classification of the zone according to its depopulation level (assigned based on the criteria of the Spanish Ministry for the Ecological Transition and the Demographic Challenge (Spanish ministry, 2023): non-depopulated, mixed, depopulated). The last variable by BHA was family medicine pressure. This variable is the average number of visits per professional per working day and represents the pressure of care exerted by the general practitioner.

### 2.3 Statistical analyses

First, the study population was described. Means and standard deviations (SD) were used to describe continuous variables, and frequencies and percentages were used to describe categorical variables. The proportions of classical and modern prescriptions were obtained (%) and described by sex. To know which individual and area characteristics were associated with being firstly prescribed a modern vs. a classical treatment, bivariate analyses were conducted. Statistical differences were assessed using chi-square (categorical variables) and Mann–Whitney tests (continuous variable).

To analyze the factors associated to a modern AD prescription, a multilevel methodology stratified by sex was developed, considering individual characteristics and characteristics of the BHA of residence. We implemented a two-level model, with a cross-classification structure, where individuals could simultaneously belong to more than one group at a given hierarchical level. In this case, we analyzed the type of AD prescription, modern or classical, by their deprivation index (quartiles), depopulation level and family medicine pressure (quartiles), so all were considered random. Cross-random effects were used when each category of one factor co-exists with each category of the other factor (there is at least one category observation for both factors).

$${VC}_{i\left(sjk\right)}=\log\frac{\pi_{sjk}}{{1-\pi }_{sjk}}=\beta_{0}+{\left(X\beta \right)}_{i\left(sjk\right)}+u_{s}+u_{j}+u_{k}+e_{i\left(sjk\right)}$$

The model had the following assumptions: first, the random effects $u_{s}$ , $u_{j}$ and $u_{k}$ were normally distributed with mean 0 and variance $\sigma_{u}^{2}$ , second, the error component $e_{i\left(sjk\right)}$ was also normally distributed with mean 0 and variance. $\sigma_{e}^{2}$ ; Third, the random effects $u_{s}$ , $u_{j}$ and $u_{k}$ and the error component $e_{i\left(sjk\right)}$ were independent, and $e_{i\left(sjk\right)}$ were all independent of each other. With i = 1, 2, n; s = 1, 2, three level of depopulation; j = 1, 2,3, 4 Quartiles deprivation index and k = 1,2,3,4 Quartiles family medicine pressure. Given the characteristics of this multilevel study, there was an intraclass correlation, which means that there were observations that are more similar to others in the same group than to those in other groups. Variance partition coefficients were calculated to see how much of the response variance belongs to each level. To evaluate statistical significance, a p-value smaller than 0.05 was used.

Interactions between variables were systematically investigated and collinearity was demonstrated. Finally, the likelihood ratio test (LR test) was used to evaluate the final model. The significance of the fixed effects was also evaluated with the Wald Test. All analyses were performed using R statistical software (the R Foundation for Statistical Computing, Vienna, Austria) using a mixed-effects linear regression based on the lme4 package (Bates et al., 2015) in the statistical package R version 4.4.0.

## 3 Results

The study population was comprised of 22,892 patients with a new T2D diagnosis, 43.52% female, mean age of 64.54 ± 13.41 years (standard deviation) (Table1). The vast majority of the population (84.68%) were native. The 54.25% of the population lived in non-depopulated areas. In terms of socioeconomic level, more than a half of the population had income of less than €18,000 per year, with differences between men and women.

**TABLE 1:** Description of the total sample analyzed and comparison by sex.

Individual variables	Total (N = 22892)	Women (N = 9963)	Men (N = 12929)	p
Age† (x̄; SD)	64.54 (13.41)	67.04 (13.77)	62.62 (12.80)	**<0.01**
Immigrant status				**0.03**
Native	19315 (84.37%)	8345 (83.77%)	10970 (84.85%)	
Immigrant	3576 (15.62%)	1617 (16.23%)	1959 (15.15%)	
Socioeconomic level				**<0.01**
Employed <18,000€ per year	4390 (19.18%)	1769 (17.76%)	2621 (20.27%)	
Employed ≥18,000 per year	2794 (12.21%)	664 (6.66%)	2130 (16.47%)	
Pensioner <18,000€ per year	8741 (38.18%)	4610 (46.27%)	4131 (31.95%)	
Pensioner ≥18,000€ per year	4262 (18.62%)	1552 (15.58%)	2710 (20.96%)	
Unemployed	1367 (5.97%)	665 (6.67%)	702 (5.43%)	
Other	1338 (5.84%)	703 (7.06%)	635 (4.91%)	
Presence of chronic morbidities				
Heart failure	1126 (4.92%)	558 (5.68%)	568 (4.45%)	**<0.01**
Chronic obstructive pulmonary disease	1532 (6.69%)	431 (4.39%)	1101 (8.62%)	**<0.01**
Depression	3425 (14.96%)	231 (23.51%)	1115 (8.73%)	**<0.01**
Ischemic heart disease	1979 (8.64%)	475 (4.83%)	1504 (11.77%)	**<0.01**
Chronic renal failure	2781 (12.15%)	1369 (13.93%)	1412 (11.05%)	**<0.01**
Dementia	569 (2.49%)	368 (3.75%)	201 (1.57%)	**<0.01**
Hypertension	12608 (55.08%)	5765 (58.68%)	6843 (53.57%)	**<0.01**
Hyperlipidemia	16916 (73.89%)	7271 (72.98%)	9645 (74.60%)	**0.01**
Obesity	5811 (25.38%)	2930 (29.66%)	2881 (22.44%)	**<0.01**
Previous MACE	746 (3.26%)	210 (62.87%)	536 (69.52%)	**0.03**
Level of prescription				
Primary care	21153 (92.40%)	9162 (91.96%)	11991 (92.74%)	**0.03**
Specialized care	1739 (7.60%)	801 (8.04%)	928 (7.26%)	
AD type				0.85
Classical	14645 (63.97%)	6583 (66.07%)	8527 (65.95%)	
Modern	8247 (36.03%)	3380 (33.93%)	4402 (34.05%)	
Analytics† (x̄; SD)				
Fasting plasma glucose	153.88 (57.20)	147.04 (53.20)	159.20 (59.59)	**<0.01**
Glycosylated hemoglobin	7.43 (1.86)	7.21 (1.69)	7.60 (1.97)	**<0.01**
Glomerular filtration rate	80.51 (18.55)	78.76 (19.35)	81.87 (17.78)	**<0.01**
BHA variables				
Depopulation level				**<0.01**
Non-depopulated	12418 (54.25%)	5675 (56.96%)	6743 (52.16%)	
Mixed	6262 (27.35%)	2611 (26.21%)	3651 (28.24%)	
Depopulated	421 (18.39%)	1677 (16.83%)	2533 (19.59%)	
Deprivation index				**0.51**
Quartile 1 (least deprived)	5587 (24.41%)	2465 (24.74%)	3122 (24.15%)	
Quartile 2	5453 (23.82%)	2366 (23.75%)	3087 (23.88%)	
Quartile 3	5006 (21.87%)	2138 (21.46%)	2868 (22.19%)	
Quartile 4 (highest deprived)	6844 (29.95%)	2994 (30.05%)	3850 (29.78%)	
Healthcare† (x̄; SD)				
Family medicine pressure	28.93 (6.30)	29.19 (6.18)	28.72 (6.38)	**<0.01**

N: number of patients; %: percentage of patients; p: statistical significance value; †Results expressed as mean (x̄) and standard deviation (SD); MACE: major adverse cardiovascular event; AD: antidiabetic drug; BHA: Basic Healthcare Area. Family medicine pressure: the average number of visits per professional per working day. Bolded numbers designate that they vary significantly from each other (p ≤ 0.05).

With regard to the presence of comorbidities, 73.89% of patients also presented hyperlipidemia, followed by 55.08% with hypertension. Women had a higher prevalence of heart failure, depression, chronic renal failure, dementia, hypertension and obesity than men. Male exhibited a higher prevalence of chronic obstructive pulmonary disease, ischemic heart disease, hyperlipidemia, and a history of previous MACE.

In consideration of the analytical values obtained prior to the initiation of the prescription, the majority of patients had elevated FPG (mean 154 ± 57.2) and HbA1c (mean 7.4%), in addition to a decreased GFR (mean 80.5 ± 18.5).

Initial treatment with monotherapy was observed in approximately 90% of cases. Metformin was the most commonly dispensed monotherapy drug, accounting for 56.88% of prescriptions, followed by combinations with metformin (12.58%); and SGLT2i (10.68%). Over one-third of patients (36.03%) started treatment with a modern AD, including DPP4i (5.92%), GLP1a (2.21%), or SGLT2i; without differences between women and men.

In Table 2, the differences between patients with a modern AD prescription and a classical one can be observed. The non-native patients and low-income people were less prescribed of modern AD. Patients with heart failure, chronic obstructive pulmonary disease, ischemic heart disease, chronic renal failure and dementia were prescribed more modern AD. Of all patients followed, 746 had had a MACE prior to the diagnosis of T2D (3.26%), of whom 415 were subsequently prescribed a modern AD. When analyzing laboratory data, in patients with poorer FPG, HbA1c and GFR control, modern drugs were prescribed more frequently. Of the prescriptions initiated in PC, the majority were for classical AD, whereas in specialized care, the majority of prescriptions were for a modern one (p = 0.00).

**TABLE 2:** Comparison of the characteristics of patients with a first prescription of a modern *vs*. classical antidiabetic drug. Bivariate analyses.

Individual variables	Classical (N = 14645)	Modern (N = 8247)	p
Age† (x̄; SD)	64.46 (12.64)	64.69 (14.69)	0.11
Immigrant status			**<0.01**
Native	2355 (16.08%)	1221 (14.81%)	
Immigrant	12289 (83.92%)	7026 (85.19%)	
Socioeconomic level			**<0.01**
Employed < 18000€ per year	2959 (19.58%)	1431 (18.39%)	
Employed ≥ 18000€ per year	1802 (11.93%)	992 (12.75%)	
Pensioner < 18000€ per year	5697 (37.70%)	3044 (39.12%)	
Pensioner ≥ 18000€ per year	2896 (19.17%)	1366 (17.55%)	
Unemployed	900 (5.96%)	467 (6.00%)	
Other	856 (5.67%)	482 (6.19%)	
Presence of chronic morbidities			
Heart failure	407 (2.81%)	719 (8.85%)	**<0.01**
Chronic obstructive pulmonary disease	916 (6.33%)	616 (7.58%)	**<0.01**
Depression	218 (15.06%)	1245 (15.32%)	0.60
Ischemic heart disease	978 (6.76%)	1001 (12.32%)	**<0.01**
Chronic renal failure	1420 (9.81%)	1361 (16.75%)	**<0.01**
Dementia	322 (2.22%)	247 (3.04%)	**<0.01**
Hypertension	8072 (55.77%)	4536 (55.83%)	0.92
Hyperlipidemia	10941 (74.71%)	5975 (72.45%)	**<0.01**
Obesity	3162 (26.95%)	1921 (31.61%)	**<0.01**
Previous MACE	331 (2.26%)	415 (5.03%)	**<0.01**
Level of prescription			
Primary care	14196 (96.93%)	6957 (84.36%)	**<0.01**
Specialized care	449 (3.07%)	1290 (15.64%)	
Analytics† (x̄; SD)			
Fasting plasma glucose	152.52 (51.83)	156.35 (65.74)	**<0.01**
Glycosylated hemoglobin	7.28 (1.67)	7.72 (2.15)	**<0.01**
Glomerular filtration rate	82.53 (16.24)	76.90 (21.61)	**<0.01**
BHA variables			
Depopulation level			**<0.01**
Non-depopulated	8184 (55.89%)	4234 (51.34%)	
Mixed	3782 (25.83%)	2480 (30.07%)	
Depopulated	2677 (18.28%)	1533 (18.59%)	
Deprivation index			**<0.01**
Quartile 1 (least deprivation)	3418 (23.34%)	2169 (26.30%)	
Quartile 2	3529 (24.10%)	1924 (23.33%)	
Quartile 3	3310 (22.60%)	1696 (20.57%)	
Quartile 4 (highest deprivation)	4386 (29.95%)	2458 (29.80%)	
Health care† (x̄; SD)			
Family medicine pressure	28.95 (6.30)	28.88 (6.29)	0.81

N: number of patients; %: percentage of patients; p: statistical significance value; †Results expressed as mean (x̄) and standard deviation (SD); MACE: major adverse cardiovascular event; AD: antidiabetic drug; BHA: Basic Healthcare Area. Family medicine pressure: the average number of visits per professional per working day. Bolded numbers designate that they vary significantly from each other (p ≤ 0.05).

Different multilevel models were tested for the total population and stratified by sex in order to know which factors were associated with the prescription of a modern AD. The model with a higher explanatory capacity was the one that combined the deprivation index, depopulation level and family medicine pressure as the BHA variables. The results of this model can be found in Table 3. In the adjusted models, we observed that the odds of prescription of modern AD decreased with age, with the group aged from 65–79 years old being the lowest one of using modern AD (odds ratio (OR) 0.48; 95% confidence interval (95%CI) 0.41–0.58). No differences were observed by sex. There were differences by socioeconomic level. So, pensioners presented the lowest probability of modern AD, being statistically significant only in women. In both sexes, patients with heart failure, ischemic heart disease, chronic renal failure, obesity and a previous MACE had more probability of having a prescription of modern AD, in addition to patients with higher FPG, HbA1c and lower GFR.

**TABLE 3:** Factors associated with the prescription of a modern antidiabetic drug. Multilevel adjusted analyses for all the population and stratified by sex.

	General Population			Women			Men		
	OR	95% CI	p	OR	95% CI	p	OR	95% CI	p
Intercept	0.88	0.67 – 1.16	0.36	1.69	1.18 – 2.42	**<0.01**	0.62	0.46 – 0.83	**<0.01**
Sex (Ref. Women)	1.01	0.93 – 1.09	0.89						
Groups of age (Ref. 16-44)									
45 - 64	0.57	0.50 – 0.66	**<0.01**	0.27	0.21 – 0.34	**<0.01**	0.85	0.71 – 1.02	0.08
65 - 79	0.48	0.41 – 0.58	**<0.01**	0.24	0.18 – 0.32	**<0.01**	0.71	0.57 – 0.89	**<0.01**
≥ 80	0.51	0.42 – 0.63	**<0.01**	0.26	0.19 – 0.36	**<0.01**	0.74	0.56 – 0.97	**0.03**
Immigration status (Ref. native)	0.79	0.71 – 0.88	**<0.01**	0.79	0.67 – 0.94	**0.01**	0.78	0.67 – 0.90	**<0.01**
Socioeconomic level (Ref. Employed <18000)									
Employed ≥ 18000	1.06	0.93 – 1.21	0.39	1.32	1.03 – 1.69	**0.03**	1.01	0.87 – 1.18	0.89
Free medicines & pensioner < 18000€	0.85	0.75 – 0.97	**0.01**	0.81	0.67 – 1.00	0.05	0.87	0.73 – 1.02	0.09
Pensioner ≥ 18000 per year	0.85	0.74 – 0.98	**0.03**	0.79	0.63 – 1.00	0.05	0.88	0.73 – 1.05	0.16
Unemployed	1.00	0.85 – 1.17	0.99	0.96	0.75 – 1.22	0.73	1.03	0.83 – 1.29	0.78
Mutualist + Other	1.14	0.97 – 1.35	0.12	1.11	0.87 – 1.41	0.39	1.16	0.92 – 1.47	0.21
Presence of chronic morbidities									
Heart failure	2.36	1.98 – 2.81	**<0.01**	2.36	1.83 – 3.04	**<0.01**	2.39	1.88 – 3.04	**<0.01**
Chronic obstructive pulmonary disease	1.13	0.98 – 1.31	0.08	1.27	0.98 – 1.66	0.07	1.05	0.88 – 1.24	0.61
Depression	1.01	0.91 – 1.12	0.88	1.03	0.90 – 1.18	0.63	1.00	0.85 – 1.18	0.98
Ischemic heart disease	1.69	1.48 – 1.92	**<0.01**	1.77	1.38 – 2.28	**<0.01**	1.59	1.37 – 1.85	**<0.01**
Chronic renal failure	1.31	1.16 – 1.48	**<0.01**	1.37	1.15 – 1.64	**<0.01**	1.25	1.06 – 1.47	**0.01**
Dementia	1.12	0.89 – 1.42	0.34	1.12	0.82 – 1.51	0.48	1.16	0.79 – 1.70	0.45
Hypertension	0.93	0.86 – 1.01	0.09	0.95	0.84 – 1.07	0.37	0.94	0.85 – 1.04	0.24
Hyperlipidemia	0.94	0.87 – 1.02	0.15	0.97	0.85 – 1.10	0.59	0.96	0.86 – 1.07	0.44
Obesity	1.29	1.19 – 1.40	**<0.01**	1.33	1.18 – 1.50	**<0.01**	1.25	1.12 – 1.40	**<0.01**
Previous MACE	1.99	1.66 – 2.39	**<0.01**	1.46	1.04 – 2.06	**0.03**	2.26	1.82 – 2.81	**<0.01**
Morbidity burden	0.96	0.92 – 1.01	0.14	0.93	0.86 – 1.00	**0.04**	1.00	0.93 – 1.07	0.94
Analytics									
Fasting plasma glucose	1.05	1.00 – 1.11	**0.04**	1.01	0.93 – 1.10	0.82	1.08	1.02 – 1.15	**0.01**
HbA1c	1.16	1.10 – 1.22	**<0.01**	1.15	1.05 – 1.26	**<0.01**	1.18	1.11 – 1.25	**<0.01**
Glomerular filtration rate	0.73	0.70 – 0.77	**<0.01**	0.71	0.66 – 0.76	**<0.01**	0.75	0.70 – 0.80	**<0.01**
Random Effects									
σ^2^	32.899			32.899			32.899		
τ_00_ Depopulation level	0.0190			0.0145			0.0198		
τ_00_ Deprivation quartile	0.0167			0.0305			0.0077		
τ_00_ Family Medicine Pressure	0.0028			0.0005			0.0027		
ICC	0.0116			0.0136			0.0091		
Observations	15338			6649			8689		
Marginal R^2^ / Conditional R^2^	0.073 / 0.083			0.088 / 0.100			0.073 / 0.081		
Deviance	18.556688			7.864772			10.611458		
AIC	18.612688			7.918772			10.665458		

OR: odds ratio adjusted by all the variables considered; 95% CI: 95% Confidence interval; p: statistical significance value; ICC: intraclass correlation coefficient; AIC: Akaike Information Criterion; MACE: major adverse cardiovascular event; Family medicine pressure: the average number of visits per professional per working day. Bolded numbers designate that they vary significantly from each other (p ≤ 0.05).

The final section of Table 3 presents the parameters associated with the model. σ2 represents the overall variance of the model, τ00 corresponds to the variances of the random effects, which are the grouping levels, ICC is the intra-class correlation index, indicating how similar the observations are within each class. In other words, it indicates that part of the total variability is due to the variability of the observations in the levels.

**FIGURE 2:**
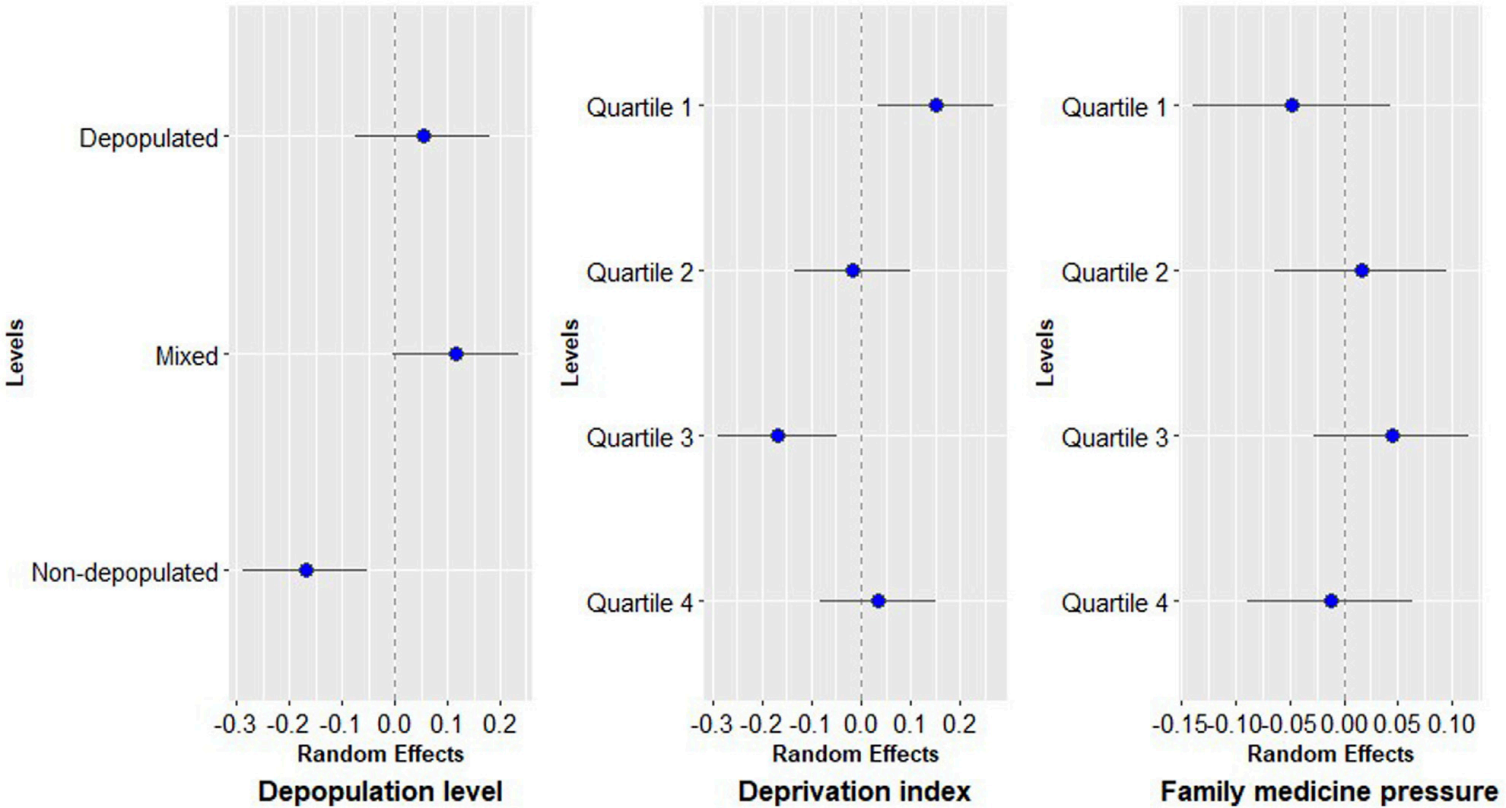
Random effects of basic healthcare area variables.

The influence of BHA variables can be observed in Figure 2. According to the depopulation level, non-depopulated areas presented the lowest risk of modern AD prescription. So, less deprived areas showed a higher probability of modern AD prescription, that decreases with the increase of deprivation until quartile 3, as is the case with family medicine pressure that increased the risk of modern AD prescription with the increasing pressure until quartile 3. There were not differences by sex as shown in Supplementary Appendix S2.

## 4 Discussion

In this population-based study conducted in patients with T2D newly diagnosed we observed differences at the individual level and by area of residence in the risk of receiving a modern AD prescription. People who were younger, had more comorbidities, higher socioeconomic level and lived in less deprived areas and depopulated areas were more likely to have a modern AD prescription.

Our study population of patients newly diagnosed with T2D were mostly male (table 1), with a mean age of 64.54, native-born and living in non-depopulated areas and with low incomes. The mean age at which diabetes was diagnosed in the subjects of our study was 64.54 years (SD = 13.41). This finding is consistent with those reported in other studies conducted in Aragón (Moreno-Juste et al., 2020) but higher than that reported in other studies both national (Mata-Cases et al., 2016; Rojo-Martínez et al., 2020) and international (Wang et al., 2019).

Our analyses revealed no significant discrepancies in the proportion of modern AD prescribed between male and female subjects. Nevertheless, the available evidence is inconclusive and exhibits inconsistencies across the studies examined (Ali et al., 2022; Choe et al., 2018) analyzing sex differences in prescribing pattern of AD. Moreover, as in our study, it has been previously described a lower prescription of SGLT2i and GLP1a in older, lower-income and immigrant populations (Ali et al., 2022; Lamprea-Montealegre et al., 2022; Montvida et al., 2018). It may be that factors such as difficulties in communication, adherence to treatment or the higher cost of modern drugs contribute to a lower frequency of prescription in these population groups (Ali et al., 2022; Lamprea-Montealegre et al., 2022; Montvida et al., 2018). Nevertheless, it is important to note that in Spain, all AD are classified as reduced-contribution treatments, whereby the patient is required to pay a fixed contribution of 10% of the retail price of the package. Furthermore, the maximum payable amount per package is set at 4.24€.

In our study, patients with comorbidities such as heart failure, ischemic heart disease, chronic renal failure, obesity and a previous MACE had a higher probability of having a prescription of modern AD. Novel AD SGLT2i and GLP1a, have shown impressive CV benefits, particularly in reducing heart failure hospitalization, CV death, and demonstrated beneficial effects on cardiac function (Nikolaidou et al., 2024). In adults with T2D, SGLT2i and GLP1a (but not DPP4i) reduce all-cause mortality and MACE compared with usual care. SGLT2i reduce chronic renal failure progression and heart failure hospitalization and GLP1a reduce stroke compared with usual care. Serious adverse events and severe hypoglycemia are less frequent with SGLT2i and GLP1a than with insulin or sulfonylureas (Qaseem et al., 2024).

In light of these considerations, clinical guidelines have been revised to designate these drugs as the first treatment option for this patient population (Davies et al., 2022). Until 2022, metformin was the primary treatment option, with treatment selection based on HbA1c levels (Bae et al., 2022). At present, comorbidities are given precedence, followed by consideration of the HbA1c level.

The majority of our patients started treatment in monotherapy and were most frequently prescribed metformin as similarly as described in a study in Catalonia (Ouchi et al., 2023). In this study also patients starting in combination therapy had higher HbA1c levels than those starting monotherapy. In clinical trials, initial combination therapy showed better glycemic control than monotherapy or a stepwise approach (Kim and Kim, 2024).

With the development of new classes of AD, the paradigm of therapeutic options for patients with glycemic and CV risk factors has shifted significantly. However, the way in which this has occurred in actual practice, especially in terms of the balance between older and newer ADs as initial and intensification therapeutic options, has undergone a much slower change (Montvida et al., 2018; Carney et al., 2022). According to a UK study (Young et al., 2023), the recent UK T2D treatment guideline represents a quasi-population indication for treatment with SGLT2i. In addition to these findings, an evaluation in the Netherlands showed that the prescription rate of SGLT2i was lower than would be indicated based on the CV risk of the patients in the absence of contraindications to their use (Hart et al., 2023). Both studies showed that these treatments were prescribed less than needed, despite being indicated for many more patients.

According to our results, the most deprived areas and non-depopulated areas presented less probability of prescription of a modern AD. Previous studies have shown that higher deprivation level is associated with higher prevalence and incidence of T2D (36,37), chronic diabetes complications, indicators of cardiometabolic risk (Laraia et al., 2012), mortality, and hospitalization due to diabetes complications (Choi et al., 2020). It is also related to worse disease control (Durfey et al., 2019) and higher treatment use (Frazer and Frazer, 2020; Di Filippo et al., 2022). It is suggested that more expensive drugs were favoured in areas with lower deprivation even for similar indications. Possible explanations include an unconscious prescriber bias towards newer and more expensive medications in affluent areas, or a more educated population likely to request newer medications (Frazer and Frazer, 2020). This phenomenon has been described for numerous chronic conditions (Di Filippo et al., 2022) and it can widen the gap in disease management and outcomes.

Regarding the multilevel model, it explained 8.5% of the variability, of which 7.3% corresponded to individual variables and 1.2% to BHA variables. It was similar when differentiating between women and men. This means that belonging to an area had very little effect on the prescription of a modern AD and that the variability was explained more by individual patient factors.

### 4.1 Strengths and limitations

The main strength of this study lies in the fact that we selected all the individuals diagnosed with T2D from 2018 to 2022, including data from administrative health data sources and electronic health records. A further key assumption is that the prescriptions we analyzed represent the majority burden of prescribing but it is important to consider that we could not collect private prescriptions. Nonetheless, they make up a minority of prescriptions, especially among chronic patients. According to the Health system review from European Observatory on Health Systems and Policies (Bernal-Delgado et al., 2024) it is described that the number of privately insured patients in 2022 in Spain only represented the 0.4% of the Spanish population aged 18 and over.

It is also necessary to take into account some limitations inherent to observational studies, such as the quality of the data, or the existence of incomplete cases. The analysis did not include diabetic patients who had been prescribed a modern drug prior to T2D diagnosis. Insidious onset of diabetes and difficulty in labelling patients with these diagnoses, may have misclassified some individuals, but this is expected to be a small number. It has also not been possible to differentiate between the different T2D subtypes. In addition, the patients included have different possible follow-up periods, ranging from 5 years to 0 days, depending on when they were diagnosed.

Modern AD have demonstrated their superiority in certain pathologies, such as heart failure, chronic renal failure or in patients with CV risk factors or obesity, to the extent that their prescription is even indicated as first-line therapy. Although these changes in the algorithms did not take place until 2023, their prescription was already more frequent with these pathologies in clinical practice. This study provides real-world evidence that the utilization pattern of AD in Patients with T2D in Aragon reflects the anticipation of clinical practice with regard to the updating of international guideline recommendations.

The findings largely reflect the prescribing patterns observed in previous research (Mata-Cases et al., 2016; Rojo-Martínez et al., 2020; Ouchi et al., 2023). The main innovation in this respect in our study is the focus on differences about deprivation index, depopulation level and family medicine pressure.

## 5 Conclusion

Sociodemographic, clinical and regional factors are associated with variability in the prescribing pattern. Our findings are helpful for understanding the use of these drugs and the predictors of their prescription, in order to better assess and understand the health outcomes of people with T2D.

The implementation of current clinical guidelines needs to be adapted to explicitly consider socio-economic and territorial factors. Although guidelines do not usually incorporate aspects such as deprivation, depopulation or care pressure, these determinants should be considered when applying them to ensure more equitable clinical practice. To this end, specific strategies such as prescription audits, targeted professional training and clinical decision support systems should be incorporated in areas with higher levels of inequality or family medicine pressure.

## Data Availability

The original contributions presented in the study are included in the article/Supplementary Material, further inquiries can be directed to the corresponding author.
