# Supplementary material for: Factors associated with prescription of modern antidiabetics in newly diagnosed patients with type 2 diabetes. a real-world data study in a Spanish region
Source: Front Pharmacol. 2025 Jul 11;16:1530139. doi: 10.3389/fphar.2025.1530139 (PMC12290405; doi:10.3389/fphar.2025.1530139)
Supplement: Supplementary file 1 [file Supplementaryfile1.docx]

Appendix 1. Drugs used in diabetes in Anatomical Therapeutic Chemical (ATC) Classification.

| **A10B - Blood glucose lowering drugs, excluding insulins** | |
| --- | --- |
| **A10BA - Biguanides** | A10BA02 - Metformin |
| **A10BB - Sulfonylureas** | A10BB M1 - Glisentide |
|  | A10BB01 - Glibenclamide |
|  | A10BB02 - Clorpropamide |
|  | A10BB03 - Tolbutamide |
|  | A10BB07 - Glipizide |
|  | A10BB08 - Gliquidone |
|  | A10BB09 - Gliclazide |
|  | A10BB12 - Glimepiride |
| **A10BD - Combinations of oral blood glucose lowering drugs** | A10BD01 - Phenformin and sulfonylureas |
|  | A10BD02 - Metformin and sulfonylureas |
|  | A10BD03 - Metformin and rosiglitazone |
|  | A10BD04 - Glimepiride and rosiglitazone |
|  | A10BD05 - Metformin and pioglitazone |
|  | A10BD06 - Glimepiride and pioglitazone |
|  | A10BD07 - Metformin and sitagliptin |
|  | A10BD08 - Metformin and vildagliptin |
|  | A10BD09 - Pioglitazone and alogliptin |
|  | A10BD10 - Metformin and saxagliptin |
|  | A10BD11 - Metformin and linagliptin |
|  | A10BD13 - Metformin and alogliptin |
|  | A10BD15 - Metformin and dapagliflozin |
|  | A10BD16 - Metformin and canagliflozin |
|  | A10BD18 - Metformin and gemigliptin |
|  | A10BD19 - Linagliptin and empagliflozin |
|  | A10BD20 - Metformin and empagliflozin |
|  | A10BD21 - Saxagliptin and dapagliflozin |
|  | A10BD23 - Metformin and ertugliflozin |
|  | A10BD24 - Sitagliptin and ertugliflozin |
| **A10BF - Alpha glucosidase inhibitors** | A10BF01 - Acarbose |
|  | A10BF02 - Miglitol |
| **A10BG - Thiazolidinediones** | A10BG02 - Rosiglitazone |
|  | A10BG03 - Pioglitazone |
| **A10BH - Dipeptidyl peptidase 4 inhibitors** | A10BH01 - Sitagliptin |
|  | A10BH02 - Vildagliptin |
|  | A10BH03 - Saxagliptin |
|  | A10BH04 - Alogliptin |
|  | A10BH05 - Linagliptin |
|  | A10BH06 - Gemigliptin |
|  | A10BH07 - Evogliptin |
|  | A10BH08 - Teneligliptin |
| **A10BJ - Glucagon-like peptide-1 analogues** | A10BJ01 - Exenatide |
|  | A10BJ02 - Liraglutide |
|  | A10BJ03 - Lixisenatide |
|  | A10BJ04 - Albiglutide |
|  | A10BJ05 - Dulaglutide |
|  | A10BJ06 - Semaglutide |
| **A10BK - Sodium-glucose co-transporter 2 inhibitors** | A10BK01 - Dapagliflozin |
|  | A10BK02 - Canagliflozin |
|  | A10BK03 - Empagliflozin |
|  | A10BK04 - Ertugliflozin |
| **A10BX - Other blood glucose lowering drugs, excl. insulins** | A10BX01 - Guar gum |
|  | A10BX02 - Repaglinide |
|  | A10BX03 - Nateglinide |
|  | A10BX06 - Benfluorex |
| **A10X - Other drugs used in diabetes** | A10X M1 - Centaurea aspera |
|  | A10XA - Aldose reductase inhibitors |
|  | A10XA01 - Tolrestat |
| **A10A - Insulins and analogues** | |
| **A10AB - Insulins and analogues for injection, fast-acting** | A10AB01 - Insulin (human) |
|  | A10AB04 - Insulin lispro |
|  | A10AB05 - Insulin aspart |
|  | A10AB06 - Insulin glulisine |
| **A10AC - Insulins and analogues for injection, intermediate-acting** | A10AC01 - Insulin (human) |
|  | A10AC04 - Insulin lispro |
| **A10AD - Insulins and analogues for injection, intermediate- or long-acting combined with fast-acting** | A10AD01 - Insulin (human) |
|  | A10AD04 - Insulin lispro |
|  | A10AD05 - Insulin aspart |
|  | A10AD06 - Insulin degludec and aspart |
| **A10AE - Insulins and analogues for injection, long-acting** | A10AE01 - Insulin (human) |
|  | A10AE04 - Insulin glargine |
|  | A10AE05 - Insulin detemir |
|  | A10AE06 - Insulin degludec |
|  | A10AE54 - Insulin glargine and lixisenatide |
|  | A10AE56 - Insulin degludec and liraglutide |
| **A10AF - Insulins and analogues for inhalation** | A10AF01 - Insulin (human) |
