# Supplementary material for: Factors associated with prescription of modern antidiabetics in newly diagnosed patients with type 2 diabetes. a real-world data study in a Spanish region
Source: Front Pharmacol. 2025 Jul 11;16:1530139. doi: 10.3389/fphar.2025.1530139 (PMC12290405; doi:10.3389/fphar.2025.1530139)
Supplement: Supplementary file 2 [file Supplementaryfile2.docx]

Appendix 2.

Figure 3. Random effects of basic healthcare area variables in women by (a) Depopulation level; (b) Deprivation index; (c) Family medicine pressure.


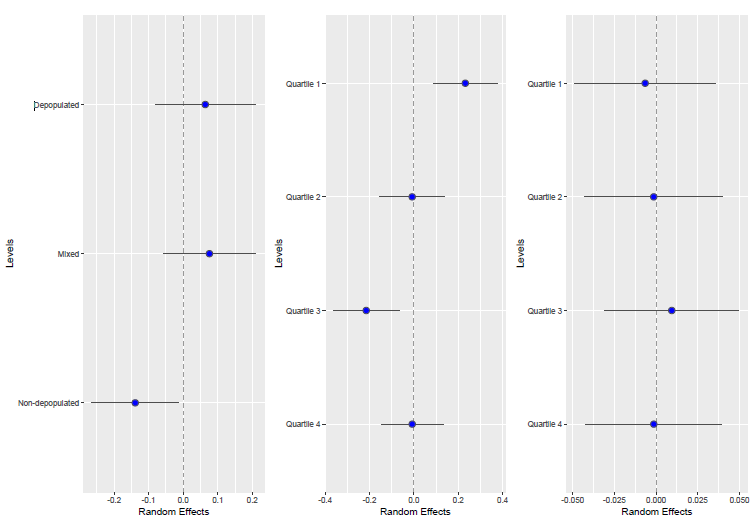


(a) (b) (c)

Figure 4. Random effects of basic healthcare area variables in men by (a) Depopulation level; (b) Deprivation index; (c) Family medicine pressure.


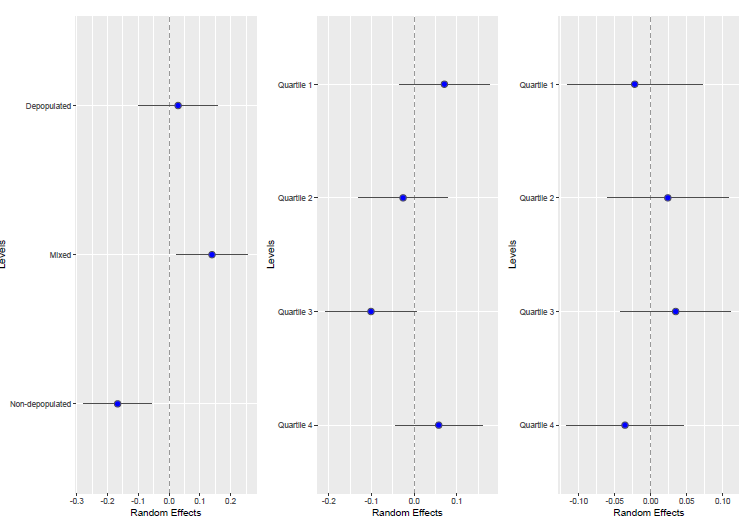


(a) (b) (c)
